# Building pathway graphs from BioPAX data in R

**DOI:** 10.12688/f1000research.9582.2

**Published:** 2016-12-12

**Authors:** Nirupama Benis, Dirkjan Schokker, Frank Kramer, Mari A. Smits, Maria Suarez-Diez

**Affiliations:** 1 Host Microbe Interactomics, Wageningen University & Research, Wageningen, Netherlands; 2 Wageningen Livestock Research, Wageningen University & Research, Wageningen, Netherlands; 3 Department of Medical Statistics, University Medical Center Goettingen, Goettingen, Germany; 4 Wageningen Bioveterinary Research, Wageningen University & Research, Wageningen, Netherlands; 5 Systems and Synthetic Biology, Wageningen University & Research, Wageningen, Netherlands

**Keywords:** rBiopaxParser, R, pathways, BioPAX

## Abstract

Biological pathways are increasingly available in the BioPAX format which uses an RDF model for data storage. One can retrieve the information in this data model in the scripting language R using the package *rBiopaxParser*, which converts the BioPAX format to one readable in R. It also has a function to build a regulatory network from the pathway information. Here we describe an extension of this function. The new function allows the user to build graphs of entire pathways, including regulated as well as non-regulated elements, and therefore provides a maximum of information. This function is available as part of the *rBiopaxParser* distribution from Bioconductor.

## Introduction

Biological pathways represent signalling and/or metabolic events involving protein and non-protein molecules. They are increasingly used in gene and protein expression studies to provide an aggregate score for gene sets encoding for defined biological events ^1^. Several pathway databases, either curated or not, have adopted the BioPAX [RRID:SCR_009881] (Biological Pathway Exchange) language as a standard for pathway representation using the RDF (Resource Description Framework) data model ^2^.

The structure of BioPAX is founded upon groupings, called classes, for physical entities and interactions with hierarchical networks of their sub-classes. Interactions between physical entities are represented such that conjoint interactions may form a specific pathway with defined, but different types of interactions between the involved physical entities. The BioPAX format is being actively developed, with BioPAX level 2 format focusing on metabolic pathways and BioPAX level 3 introducing full support for signalling pathways.

SPARQL (Simple Protocol And RDF Query Language) is a query language able to retrieve and manipulate data stored in RDF. Pathway information is often combined with statistical data analysis using tools such as R ^3^. The *rBiopaxParser* [RRID:SCR_002744] ^4^ is an R package to retrieve data stored in a BioPAX RDF format. It comes with several options that are useful to probe the data and extract specific information from it, for example participants of a pathway, stoichiometric conditions to be fulfilled for an interaction, etc.

**Figure 1.**
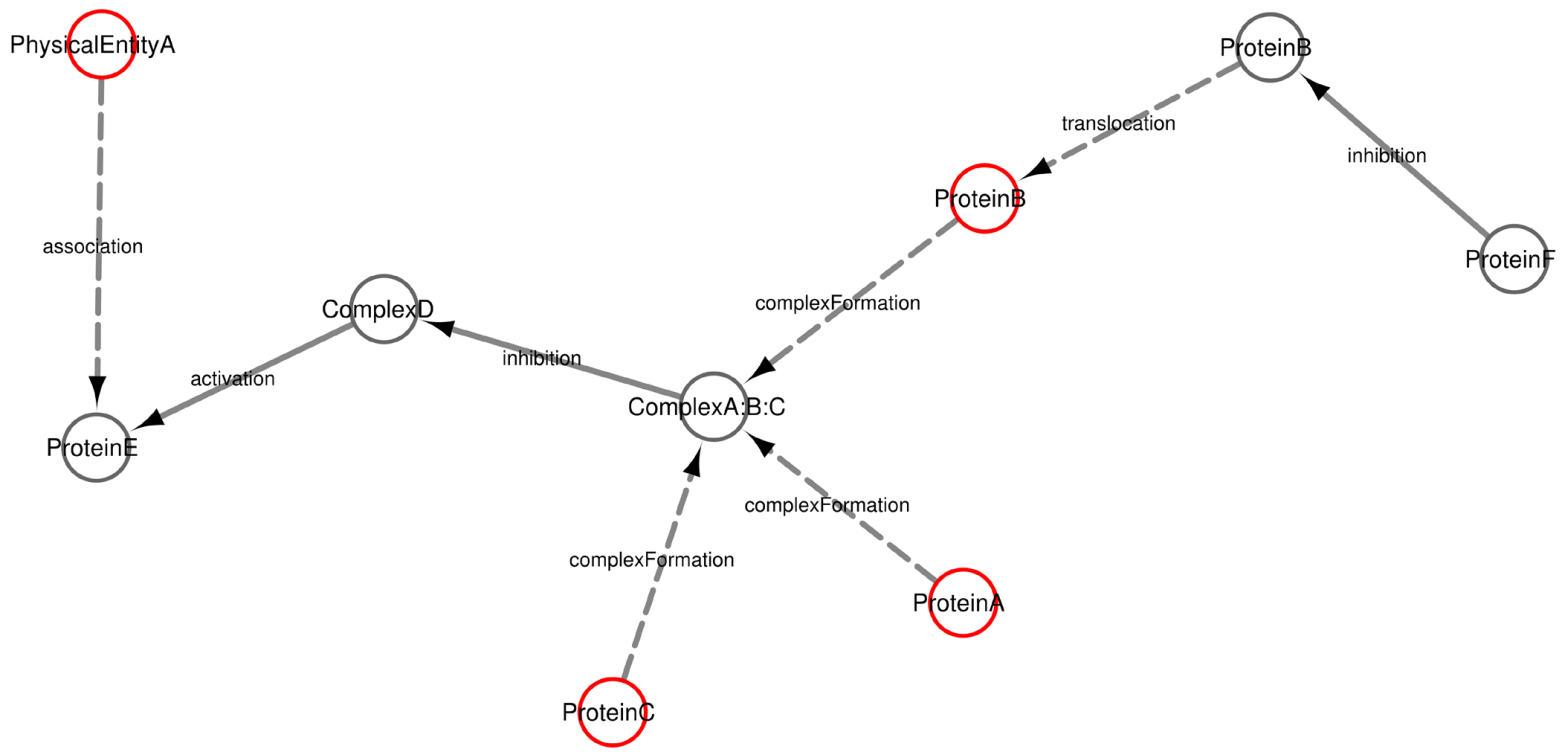
Hypothetical pathway. This cartoon of a pathway shows examples of nodes and edges that could be encountered in a BioPAX database. The nodes are proteins, complexes or other physical entities and the edges are interactions between the nodes, that represent either interactions among proteins or protein modifications. The solid edges are those detected by the P2RG function and the solid and dashed edges are detected by the P2G function.

One such option is the *pathway2RegulatoryGraph* (P2RG) function that converts a pathway into a graphical structure. This is extremely useful for visual representation and subsequent graph-based network analysis. The P2RG function returns the parts of a pathway that are regulated (activated or inhibited) by proteins or protein complexes; this is important to understand the role of regulated proteins in pathways. Here we present an adaptation of P2RG, denoted *pathway2Graph* (P2G) which can be used to build a graph of the entire pathway, including the regulated as well as the non-regulated elements. This new function expands P2RG and can be used to investigate all different types of processes and connections of pathways instead of only studying the regulated elements of pathways. P2RG retrieves regulatory interactions, such as inhibitions and activations (shown in Figure 1 as continuous edges). The new P2G additionally, retrieves protein modifications, such as translocations or complex formation, which are shown as discontinuous edges in Figure 1.

P2G is specifically aimed at retrieving results from Reactome BioPAX level 3. In this paper we describe detailed information on this function which, we believe, will help rBiopaxParser users to better understand the graphs generated from pathway information. We have verified P2G results by directly querying the original BioPAX data using SPARQL.

## Methods and results

**Figure 2.**
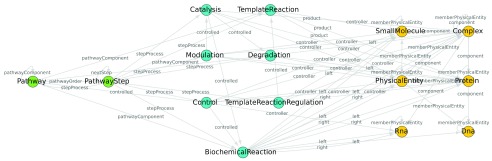
Interplay of classes in Reactome BioPAX. This figure shows a network of the Interaction and PhysicalEntity classes that are a part of any pathway in Reactome v51 BioPAX level 3. Nodes are classes and the directed edges are links between them in the database. The green nodes are the Pathway and PathwayStep classes, the blue nodes are Interaction classes and orange nodes are PhysicalEntity classes.

The classes of **PhysicalEntity** and **Interaction** that are used in Reactome v51 to represent information on pathways are shown in Figure 2. This graph was generated using the tool RDF2Graph ^5^ on the Reactome Level 3 RDF file. The nodes in Figure 2 represent classes and the edges show the possible relationships, called predicates, these classes could have in the database. As depicted in Figure 2, the node **Pathway** could have one or more **PathwaySteps** that consist of different types of **Interaction** sub-classes. All the **Interaction** nodes shown in Figure 2 describe interactions between **PhysicalEntities**, hence are connected to them by particular types of predicates as indicated in the edge labels. The **Interaction** classes are interconnected because they can be dependent on each other. The **Control** interaction and its sub-classes ( **Catalysis** and **Modulation**) represent signalling events. They regulate **BiochemicalReaction** and **Degradation** interactions which mostly represent metabolic reactions.

To create a regulatory graph, the P2RG function starts with the **Control**, **Catalysis** and **Modulation** interactions that are either activating or inhibiting other interactions. This method provides a graph with plenty of information on the regulatory components of the pathway. The nodes of this graph are physical entities like **Proteins** or **SmallMolecules** and the directed edges are either activation or inhibition events. An example of such a reconstruction is shown in Figure 1, where P2RG is able to retrieve the black nodes and the continuous edges. However, interactions can be missed if they are not regulated by the **Control** interactions and could result in the loss of valuable information in the graphical representation of the pathway.

The new function P2G can start with any type of interaction in order to obtain a graph with all possible physical entities involved in the pathway. Similar to the result of the P2RG function, the P2G function gives a graph with nodes that are physical entities, but the edges are not strictly activation or inhibition events. The directed edges could represent several types of events like translocation of a protein or cleavage of DNA, these are shown as dis-continuous edges in the cartoon in Figure 1. The P2G function recognizes the continuous and the dis-continuous edges and thus retrieves the black as well as the red nodes shown in Figure 1. In some cases there is more than one documented connection between the same physical entities. In this case only the first connection is used as an edge in the final pathway graph.

## Comparison of two methods: P2G vs P2RG

**Figure 3.**
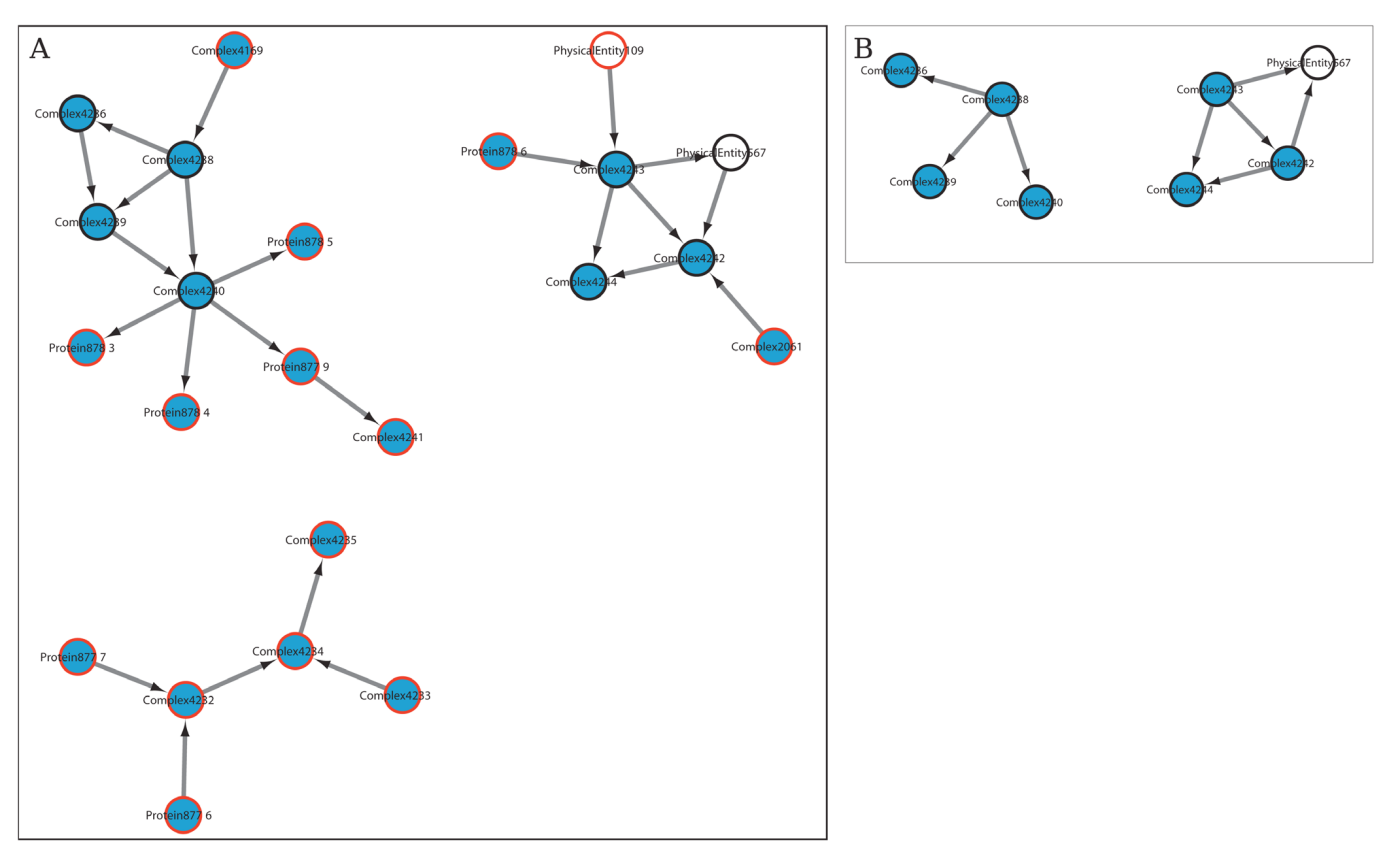
Graphs of the pathway ‘Apoptosis induced DNA fragmentation’. Both graphs were extracted from the same BioPAX file. **A**) Graph recovered using the new P2G function; **B**) Graph recovered using P2RG function. In both panels blue nodes are proteins or protein complexes, white nodes are non-protein entities. Black encircled nodes are found in both graphs and red encircled nodes are only detected with the new P2G function. Names of the nodes are in Table 2.

The Reactome database (v51) categorizes pathways into 27 branches. Here we worked with pathways that have more than one interaction, which resulted in 1,666 pathways. Using P2RG, graphs for 1,548 pathways were retrieved. By using the new P2G function, we were able to retrieve information on all 1,666 pathways. The highest number of pathways were obtained, using either method, in the “Disease” category (P2RG: 3,396 pathways, P2G: 4,888 pathways). In 85% of the cases, pathways retrieved using P2G consisted of more physical entities (nodes) than those retrieved using P2RG. 19% of the P2G retrieved pathways have at least twice the number of nodes, and 60% have at least twice the number of interactions between nodes (edges) as compared to the P2RG version, Figure 3 is an example of this difference. Total numbers of nodes and edges in major Reactome categories are given in Table 1. Missing information causes the appearance of disconnected graphs when reconstructing pathways. By using the new P2G function, the percentage of disconnected pathways is reduced by 9%. Additionally, P2G also has the option of only retrieving the largest connected component, for example with this option, in Figure 3. A only the top left part of the graph will be retrieved and the two other disconnected parts discarded. The pathways have directed edges because most of the interactions have direction. Edges without a direction are represented as bidirectional edges in the output of P2G.

**Table 1.** Numbers of nodes and edges. The number of nodes and edges of ten different pathways (Reactome Categories) are indicated as obtained after application of P2RG and P2G on the same set of BioPAX RDF information.

Reactome Categories	P2RG Nodes	P2RG Edges	P2G Nodes	P2G Edges
Binding and Uptake of Ligands by Scavenger Receptors	0	0	68	56
Cell-Cell communication	13	14	142	142
Disease	3,396	5,878	4,888	12,159
Gene Expression	652	900	1,110	2,450
Immune System	1,431	2,233	2,419	5,045
Membrane Trafficking	86	121	181	382
Metabolism	3,082	5,922	3,479	11,289
Signaling Pathways	2,069	3,274	3,430	7,131
Steroid hormones	72	147	81	333
Transcription	281	420	623	1,324

As an example, we discuss here the ‘Apoptosis induced DNA fragmentation’ pathway, which is in the “Programmed Cell Death’ category ( Figure 3). When the information in the BioPAX file is reconstructed with the P2RG function, the pathway has seven nodes ( Figure 3.B); with the P2G function the same pathway has 16 more nodes ( Figure 3.A). Detailed information on these nodes, as retrieved with P2G and P2RG, is given in Table 2 and demonstrates the additional information retrieved by P2G. The node ‘Complex4169’, which is found in the cytosol, translocates to the nucleus where it is called ‘Complex4238’. However, this information is only available from the P2G function because the node ‘Complex4169’ does not regulate any other interaction or node. The presence of extra nodes in the P2G retrieved graph ( Figure 3) also visualizes that ‘Complex4240’ breaks up into its’ individual components after being cleaved by Caspase-3 (‘Complex4238’). This extra information is very useful for researchers analysing the phenomena represented by the pathway. In case P2G retrieved pathways graphs are used for analysis (e.g, differential gene expression analysis) the presence of these extra nodes may improve biological interpretation of experimental data.

**Table 2.** Node names and locations of the “Apoptosis induced DNA fragmentation” pathway. The first column has the names of the nodes in the pathwayas depicted in Figure 3. The second column has the actual name of the node and the third column the cellular location of the node. All this information is represented as given in Reactome version 51. The nodes shown with a black outline in Figure 3 are shown here in bold font.

Node	Name	Location
Protein8776	DFFB	Cytosol
Protein8777	DFFA	Cytosol
Complex4232	DFFA : DFFB	Cytosol
Complex4233	Importin alpha : Importin beta	Cytosol
Complex4234	DFF : associated with Importin alpha : Importin beta	Cytosol
Complex4235	DFF : associated with Importin alpha : Importin beta	Nucleoplasm
Complex4169	Active CASP3	Cytosol
**Complex4238**	**Active CASP3**	Nucleoplasm
**Complex4236**	**DFFA : DFFB**	Nucleoplasm
**Complex4239**	**Caspase cleaved DFFA**	Nucleoplasm
**Complex4240**	**Caspase cleaved DFFA : DFFB**	Nucleoplasm
Protein8779	DFFB	Nucleoplasm
Protein8784	DFFA fragment	Nucleoplasm
Protein8785	DFFA fragment	Nucleoplasm
Protein8783	DFFA fragment	Nucleoplasm
Complex4241	DFFB homodimer	Nucleoplasm
**PhysicalEntity567**	**DFFB homodimer/homooligomer**	Nucleoplasm
Complex2061	Histone H1 bound chromatin DNA	Nucleoplasm
**Complex4242**	**DFFB associated with chromatin**	Nucleoplasm
Protein8786	HMGB1/HMGB2	Nucleoplasm
PhysicalEntity109	DNA	Nucleoplasm
**Complex4243**	**HMGB1/HMGB2 – bound chromatin**	Nucleoplasm
**Complex4244**	**DFF cleaved DNA**	Nucleoplasm

## Conclusion

P2G is a useful addition to the rBiopaxParser package because it retrieves all the components of a pathway from the database and provides complete graphical information for both signalling as well as metabolic pathways. The P2G function ( *pathway2Graph*) is currently available in the *rBiopaxParser* package in the Bioconductor 3.4 release.

## Data availability

The data referenced by this article are under copyright with the following copyright statement: Copyright: © 2016 Benis N et al.

The input data for this package is the BioPAX format of any pathway database. We used the Reactome database which is freely available for download in different formats from the website www.reactome.org. A subset of this database is given as Supplementary file 1.

## Software availability

**Software available from**: The function pathway2Graph is available in the latest version of the R package *rBiopaxParser* and can be installed from Bioconductor.

**Latest source code**: https://github.com/frankkramer-lab/rBiopaxParser/tree/2.12.0

**Archived source code as at the time of publication**: http://dx.doi.org/10.5281/zenodo.61618 ^6^

**Software license**: GPL-2

## Supplementary material

**Subset of Reactome database.**

This .owl file contains information on four pathways from the Reactome v51 BioPAX level 3 database. This format can be loaded into the R environment using the rBiopaxParser package and used to test the P2G function and obtain graphs which were used as the basis for Figure 3. More information on loading and processing this file format can be found in the package documentation.
