# Supplementary material for: Building pathway graphs from BioPAX data in R
Source: F1000Res. 2016 Dec 12;5:2414. Originally published 2016 Sep 28. [Version 2] doi: 10.12688/f1000research.9582.2 (PMC5130076; doi:10.12688/f1000research.9582.2)
Supplement: Peer review history

# Peer review: Referee response for version 2

**DOI:** 10.5256/f1000research.11188.r18423

**Published:** 2016-12-13

**Authors:** Stephen N. Floor

**Affiliations:** 1 Department of Molecular and Cell Biology, University of California, Berkeley, Berkeley, CA, USA

The authors have addressed the (minor) issues I had with the article previously, thanks!

I have read this submission. I believe that I have an appropriate level of expertise to confirm that it is of an acceptable scientific standard.

# Peer review: Referee response for version 1

**DOI:** 10.5256/f1000research.10320.r17476

**Published:** 2016-12-02

**Authors:** Kyle Ellrott

**Affiliations:** 1 Oregon Health & Science University, Portland, OR, USA

The authors of this paper describe a new function provided by the rBiopaxParser library, which is a R based system for parsing BioPax documents. BioPax is coded in RDF, which is a linked data format that describes the subject matter using graph triples in the form of a Subject, Predicate and Object. Their pathway2Graph and the old gathwayRegulatoryGraph essentially attempt to do graph transformations, taking the RDF graph and converting it to a graph that is suitable for analysis. 

 The paper’s primary metric for demonstrating improvements in their method is measuring the number of non-zero pathways retrieved after extracting data from Reactome. In addition, I tested code and their supplied supplemental data. 

 ```

 > library(rBiopaxParser)

 > biopax=readBiopax("19c8ac7b-96b2-4db4-a78a-c2defed535ae.owl")

 > a <- pathway2RegulatoryGraph(biopax, "Pathway1020")

 > b <- pathway2Graph(biopax, "Pathway1020")

 > nodes(a)

  [1] "CASP3"                        "CASP3(176-277)"

  [3] "DFFA(1-?)"                    "DFFB"

  [5] "DFFA(118-331)"                "DFFA(1-117)"

  [7] "DFFA(225-331)"                "DFFA(118-224)"

  [9] "Histone H1"                   "DFF40 homodimer/homooligomer"

 [11] "HMGB1/HMGB2"

 > nodes(b)

  [1] "Protein8776"       "Protein8777"       "Complex4232"

  [4] "Complex4233"       "Complex4234"       "Complex4235"

  [7] "Complex4169"       "Complex4238"       "Complex4236"

 [10] "Complex4239"       "Complex4240"       "Protein8779"

 [13] "Protein8784"       "Protein8785"       "Protein8783"

 [16] "Complex4241"       "PhysicalEntity567" "Complex2061"

 [19] "Complex4242"       "Protein8786"       "PhysicalEntity109"

 [22] "Complex4243"       "Complex4244"

 ```

 From the old version (a) to the new extraction (b), for this particular pathway the number of nodes went from 11 to 23. Interestingly, the names of the vertices became less descriptive in the newer method, going from gene names like ‘CASP3’ to strings extracted from the RDF urls, like ‘Protein8776’.

 It would seem that the authors claim, that the number of extracted elements from the BioPax files is accurate. But what is slightly confusing is the significance of this change. Was the original method faulty and this is a bug fix? Or is the method being updated to deal with the BioPax standard as it changes from version 1 to 2 to 3? 

 This seems like a minor but necessary change to the library to make it have a better extract information from BioPax. More explanation about the nature of this change, and how the new parsing strategy improved how the library was able to deal with all of the various types of relationship classes in the BioPax format would make a big difference in helping to illustrate the improvements mentioned in this paper.

I have read this submission. I believe that I have an appropriate level of expertise to confirm that it is of an acceptable scientific standard, however I have significant reservations, as outlined above.

# Peer review: Referee response for version 1

**DOI:** 10.5256/f1000research.10320.r17940

**Published:** 2016-11-24

**Authors:** Hilary Ann Coller

**Affiliations:** 1 Department of Molecular, Cell and Developmental Biology, University of California Los Angeles, Los Angeles, CA, USA

The authors have developed a new function that allows the user to build a regulatory network in a graph format based on pathway information. In the version that the authors developed, the output graph includes regulatory and non-regulatory interactions and allows the viewer to more fully comprehend the underlying network. Nodes in the network represent classes, while the edges show the relationships among these classes. An example of the approach is provided with the Reactome database. The authors’ approach, P2G, was used to analyze data for 1,666 pathways. P2G returned more nodes than was retrieved by the earlier version in 85% of the cases. The software will be available as part of the Bioconductor 3.4 release. This will likely be a valuable addition to the Bioconductor package that will provide scientists with a means for generating graphical and intuitive networks from gene expression and metabolic data. The manuscript is clear with an appropriate title and abstract. The article is clearly written and the conclusions are based on the data.

I have read this submission. I believe that I have an appropriate level of expertise to confirm that it is of an acceptable scientific standard.

# Response

**Published:** 2016-11-30

**Authors:** Nirupama Benis

**Affiliations:** Host Microbe Interactomics, Wageningen University and Research, Netherlands

Thank you for your comments.

# Peer review: Referee response for version 1

**DOI:** 10.5256/f1000research.10320.r17305

**Published:** 2016-11-07

**Authors:** Stephen N. Floor

**Affiliations:** 1 Department of Molecular and Cell Biology, University of California, Berkeley, Berkeley, CA, USA

The authors have developed a new function in the *rBiopaxParser *package to generate figures from BioPAX formatted biological pathway data. This new function, called *pathway2Graph* (P2G) replaces an older function called *pathway2RegulatoryGraph* (P2RG). P2G includes more interaction terms than P2RG, and therefore generates more complete interaction graphs. That said, as written the changes are mysterious - why did the original function limit the interaction types when generating graphs and what is advantageous about including more interactions? Addition of the P2G function is a small improvement to the rBiopaxParser package that will be useful, but the discussion of P2G's advantages and disadvantages should be expanded.

 Specific points:

1. The language throughout is highly technical, potentially compromising its readability to end users.
2. The edge labels in Figure 1 are very difficult to read. As these are discussed in the text, their font size and/or weight should be increased.
3. It would increase the readability of this software tool article if the authors described a (biological) scenario where the P2G function would be uniquely useful compared to P2RG. It’s obvious that including more interaction types will lead to more complete graphs, but in what scenario would this be useful for a user?
4. Why were all interaction types not originally included in P2RG? The advantages and any potential disadvantages of including all interaction types should be discussed more.
5. Currently, the difference between P2G and P2RG is rather minor (1,666 vs 1,548) – might this difference change in the future if more edges are added through the interaction types that are unique to P2G?

The work is technically sound and presents a useful extension to the rBiopaxParser package, but the paper describing this work will be useful to a broader audience if changes similar to those above are incorporated.

I have read this submission. I believe that I have an appropriate level of expertise to confirm that it is of an acceptable scientific standard, however I have significant reservations, as outlined above.

# Response

**Published:** 2016-11-30

**Authors:** Nirupama Benis

**Affiliations:** Host Microbe Interactomics, Wageningen University and Research, Netherlands

Thank you for your comments. In the new version we have expanded the Introduction with non-technical specifics that should explain the basic differences between the functions to a broader audience. Figure 2 (Figure 1 in the previous version) has been changed to increase visibility of edge labels. In order to explain the differences between the outputs of the two functions we have added a section in the Methods and Results section where we describe a particular pathway (‘Apoptosis induced DNA fragmentation’) in terms of the extra information gained by using the new function. A new table (Table 2) with more biological information on this pathway has also been added.

# Peer review: Referee response for version 1

**DOI:** 10.5256/f1000research.10320.r17096

**Published:** 2016-11-02

**Authors:** Lynn Fink

**Affiliations:** 1 University of Queensland, Diamantina Institute, Woolloongabba, QLD, Australia

This articles describes the addition of a new function to the extant rBiopaxParser R library. This new function converts a BioPAX-formatted pathway of gene or protein interactions into a graphical structure that is human-viewable. This function supercedes an earlier function which performed the same task, but was unable to convert all nodes and egdes in the pathway. This resulted in a loss of information. This loss is now remedied, however, with the new and improved function.

 I installed the package and tested it and it seemed to work flawlessly, although I didn't try anything fancy. I imagine that the new function is a significant advance for people working on these pathways routinely as it must have been frustrating to have missing data.

 My only reservation about the paper is that I was unclear on the point of the new function until I'd read most of the paper. Perhaps the authors could extend the last paragraph of the introduction to be more clear about why the new function was necessary and how it has improved the R package. Although this request is a small change, the article is confusing as it is and making this would be a big improvement.

I have read this submission. I believe that I have an appropriate level of expertise to confirm that it is of an acceptable scientific standard, however I have significant reservations, as outlined above.

# Response

**Published:** 2016-11-30

**Authors:** Nirupama Benis

**Affiliations:** Host Microbe Interactomics, Wageningen University and Research, Netherlands

Thank you for your comments. The Introduction has been expanded with an image (now Figure 1) to emphasize the differences between the two functions.
